# Rising rates of sepsis in England: an ecological study

**DOI:** 10.1007/s15010-025-02601-0

**Published:** 2025-07-15

**Authors:** Victoria B. Allen, Katie Bechman, Mark D. Russell, Maryam A. Adas, Anna L. Goodman, Mark J. McPhail, Sam Norton, James B. Galloway

**Affiliations:** 1 Centre for Rheumatic Diseases, King’s College London, London, UK; 2 Department of Infection, King’s College London, London, UK; 3 Department of Infection, Guy’s and St Thomas’ NHS Foundation Trust, London, UK; 4 MRC Trials Unit, University College London, London, UK; 5 Institute of Liver Studies, King’s College London, London, UK

**Keywords:** Sepsis, Public health, Routinely collected health data, Incidence, Length of stay

## Abstract

**Purpose:**

Sepsis is life-threatening organ dysfunction caused by a dysregulated host response to infection. It is a major cause of morbidity and mortality. A contemporary overview of sepsis epidemiology in England is long overdue. This study provides an update on the incidence of sepsis-coded hospital admissions and mortality following the COVID-19 pandemic, focusing on the relative contribution of different bacterial pathogens to sepsis-coded admissions.

**Methods:**

We undertook a descriptive study of all hospital admissions from April 1998 to March 2024 using routinely collected health data. Information on sepsis admission episodes, causative pathogens, age, sex, length-of-stay and mortality were collected.

**Results:**

Sepsis-coded hospital admissions increased from 27.9 admissions per 100,000 in 1998 to 210.4 in 2023, a 7.5-fold increase. The incidence of sepsis-coded admissions due to most pre-specified pathogens of interest increased. The largest increases were seen for sepsis due to *Enterococci*, *Streptococcus pyogenes*, gram-negative bacteria, *Streptococcus agalactiae*,* Staphylococcus aureus* and *Listeria spp*. Sepsis due to meningococcus decreased. The percentage of patients aged ≥ 75 years admitted with sepsis increased from 32.4 to 52.5% of sepsis cases. Median length-of-stay was 6.1 days. Sepsis-coded admissions and mortality decreased during the COVID-19 pandemic. These have now returned to pre-pandemic levels.

**Conclusion:**

The recorded incidence of sepsis-coded hospital admissions has risen. This may have been impacted by coding changes and improved disease recognition. The decrease in meningococcal sepsis may reflect the success of vaccination campaigns. Further research is needed to explore concurrent trends in sepsis severity, predict who is at greatest risk and improve prevention efforts.

**Supplementary Information:**

The online version contains supplementary material available at 10.1007/s15010-025-02601-0.

## Introduction

Sepsis is defined as “life-threatening organ dysfunction caused by a dysregulated host response to infection.” [1] It is a major cause of morbidity and mortality with approximately 50 million cases worldwide in 2017 leading to an estimated 11 million deaths [2]. Sepsis is also an important public health concern more locally in England and Wales. There were an estimated 200,000 admissions to hospitals in England due to sepsis in 2017 [3]. The death rate from sepsis in England has been estimated to be approximately 20% [4] and sepsis accounts for around a third of admissions to adult general intensive care units (ICUs) [5].

The reported incidence rate of sepsis has been rising over recent decades, both in England and internationally [5–9]. However, few studies have reported on epidemiological trends in sepsis incidence in England in recent years, particularly following the COVID-19 pandemic. Various clinical factors have been cited as possible explanations for rising sepsis incidence, including aging populations, increased use of immunosuppression, more invasive procedures and rising antimicrobial resistance [10]. There has also been increasing awareness of the importance of timely diagnosis and management of sepsis within healthcare settings [11], through various initiatives including the Surviving Sepsis Campaign [12] which may have impacted recorded diagnoses of sepsis.

Sepsis can be caused by a wide range of underlying infectious pathogens. These are most commonly bacterial but can also be fungal, viral or parasitic. The United Kingdom Health Security Agency (UKHSA) carries out surveillance of certain specified bacteraemias but the relative contribution of these and other bacterial pathogens to hospital admissions due to sepsis has not been investigated. Understanding which pathogens are driving sepsis admissions is vital for public health efforts, effective antimicrobial prescribing and infection prevention and control strategies.

The onset of the COVID-19 pandemic was associated with a decline in hospital admissions due to non-COVID sepsis in England [13] and other countries such as Denmark [14] and Norway [15]. Trends in hospital admissions due to sepsis and sepsis-mortality following the pandemic have not been described. An updated picture of sepsis epidemiology is essential for assessing whether sepsis incidence has continued to increase in England.

The National Health Service (NHS) in England curates an administrative dataset called Hospital Episode Statistics Admitted Patient Care (HES-APC). This dataset contains details on hospital admissions to NHS-commissioned hospital services in England accounting for 98–99% of all hospital activity in England [16]. This dataset offers near-universal coverage and a long period of data collection, making it a useful resource for examining epidemiological trends in admissions due to sepsis, with the important caveat that such administrative, coding data may not always accurately capture clinical reality. Despite this, the accuracy of routinely-collected health data is improving and data support their use for research purposes [17].

This study aims to update contemporary sepsis epidemiology in England. Our primary objective is to report trends in hospital admissions due to sepsis for the years before, during and after the COVID-19 pandemic. Our secondary objectives are to describe trends in hospital admissions due to sepsis caused by particular pathogens of interest and to describe trends in mortality due to sepsis in England and Wales.

## Methods

### Study type

We conducted a population-level, descriptive study of sepsis-coded hospital admissions in England and of death registrations due to sepsis in England and Wales.

### Data sources

Hospital episode data were obtained from HES-APC Records. These form part of the Hospital Episode Statistics (HES) database published annually in aggregated form by NHS Digital and are freely available [18]. Anonymised data for all hospitalisations in NHS hospitals in England are summarised according to International Classification of Diseases, 10th revision (ICD-10) codes for primary and secondary admission diagnoses. Data on age and median length of hospital stay are also available. A year runs from April 1st to March 31st and all NHS hospitals in England are included. For narrative clarity, only the start year of each 12-month period will be referred to within the text of this paper, e.g. April 1st 1998 to March 31st 1999 will be referred to as 1998. Mid-year population estimates for England were used in the analysis of hospital admissions. These are published by the Office for National Statistics (ONS) and are publicly available [19].

Data on death registrations due to sepsis used data from England and Wales published by the ONS [20]. A year runs from January to December. Mid-year population estimates for England and Wales were used in the analysis of mortality due to sepsis. These were obtained from the same source as the mid-year population estimates above.

### Study period

The study periods for each analysis were chosen to include all published datasets available at the time of conducting the study. This corresponded to April 1st 1998 to March 31st 2024 for the analysis of hospital admissions due to sepsis and the calendar years 2001 to 2023 for the analysis of death registrations due to sepsis.

### Incidence of sepsis-coded hospital admissions

The number of admissions with a primary diagnosis of sepsis were divided by the mid-year population estimate and reported as an incidence per 100,000 of the population. A list of ICD-10 codes used to identify sepsis-coded admissions is included in the supplementary material. Codes were chosen based on clinical relevance and availability within ICD-10. The majority of available codes were either non-specific or bacterial although where available fungal and viral codes have been included.

The percentage of sepsis-coded admissions across different age groups was calculated. Ages were grouped into the following categories, 0–14, 15–59, 60–74 and 75 + based on the categories used in the HES data. The percentages of admissions in male and female patients were calculated along with the median length of hospital stay in days.

### Incidence of sepsis-coded admissions due to specific bacterial pathogens of interest

Sepsis-coded hospital admissions due to specific bacterial pathogens were identified from HES data using pre-specified ICD-10 codes. Microbiological confirmation was not available. Pre-specified sepsis codes were chosen on the basis of clinical relevance and availability of specific ICD-10 codes. These codes included sepsis due to *Staphylococcus aureus*, Group A streptococcus (*Streptococcus pyogenes*), Group B streptococcus (*Streptococcus agalactiae*), Group D streptococcus (*Streptococcus bovis/Streptococcus equinus* complex) and enterococci, *Streptococcus pneumoniae*, *Listeria spp*., Non-typhoidal Salmonella, *Haemophilus influenzae* and *Neisseria meningitidis*. The pre-specified codes also included a “sepsis due to other Gram-negative organisms” code. This code may be used for Gram-negative organisms not listed above (E.g., *Escherichia coli*,* Pseudomonas aeruginosa* etc.).

The incidence of sepsis-coded admissions due to specific bacterial pathogens of interest was calculated as the number of hospital admissions including a primary diagnosis of sepsis due to each specific pathogen, per 100,000 of the population in England, for each year of the study period.

### Mortality rates

Mortality rates were calculated using the number of death registrations in England and Wales. Death due to sepsis was defined as either sepsis mentioned anywhere on the death certificate or as the main, “underlying” cause of death. The ONS define the “underlying” cause of death as the particular condition that causes the death [21]. The ONS defined sepsis using ICD-10 codes A40-A41, A39.2, A39.3 and A39.4 (see supplementary material for descriptive terms). Mortality rates were calculated per 100,000 of the population of England and Wales.

### Statistical analysis

Data were visualised using scatter plots with locally weighted scatterplot smoothing (LOWESS) curves. Linear regression was performed to examine trends in the annual incidence of hospital admissions with a primary diagnosis of sepsis over the study period. The Ramsey RESET test and inclusion of a quadratic term were used to assess model specification and potential non-linearity. Additionally restricted cubic splines and Joinpoint analysis were applied to explore potential non-linear trends. Trends in the annual incidence of hospital admissions due to sepsis caused by individual pathogens of interest were analysed descriptively. Weighted averages for age, sex and median length of stay were calculated using count frequencies for relevant codes. Statistical analyses were performed using Stata/MP 18.0 (StataCorp).

### Ethics

Ethical approval was not required as all data are in the public domain and are anonymised and aggregated.

## Results

### Incidence of hospital admissions due to Sepsis

The annual incidence of hospital admissions with a primary coded diagnosis of sepsis between April 1998 and March 2024 is shown in Table 1. Across this period the reported incidence of sepsis-coded admissions increased from 27.9 admissions per 100,000 population in 1998 to 210.4 admissions per 100,000 population in 2023 representing a 7.5-fold increase. Extrapolating these figures suggests there were approximately 121,000 sepsis-coded hospital admissions in 2023. Hospital admissions due to sepsis as a proportion of all hospital admissions increased from 123.7 admissions per 100,000 to 691.2 in the same period representing a 5.6-fold increase.

**Table 1:**
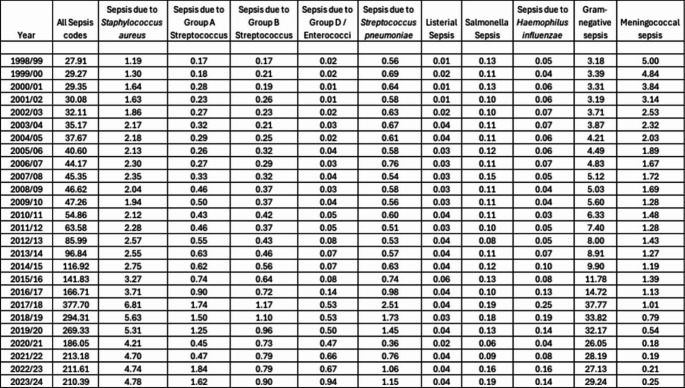
Annual incidence of sepsis-coded hospital admissions between April 1998 and March 2024. Admissions are reported for all sepsis codes combined and for sepsis due to specific pathogens of interest. (Note “Gram-negative sepsis” contains multiple organisms). Admissions are reported per 100,000 of the population of England using ONS mid-year population estimates

Hospital admissions due to sepsis increased gradually between 1998 and 2010, increasing approximately two-fold over this twelve-year period *(see* Fig. 1a*).* A steeper rise was seen over the six-year period from 2010 to 2016, during which sepsis-coded admissions increased approximately three-fold. Admissions increased sharply in 2017, more than doubling from the previous year. This coincided with national guidance that changed the way sepsis was coded in England and Wales. Admissions decreased by 22% in 2018 following further changes to national coding guidance. In 2020 admissions decreased by 31% coinciding with the first and second waves of the COVID-19 pandemic (see supplementary material Fig. 1). Sepsis-coded admissions then increased by 15% in 2021 and have stabilised at that level in the years following the pandemic.

**Fig. 1a:**
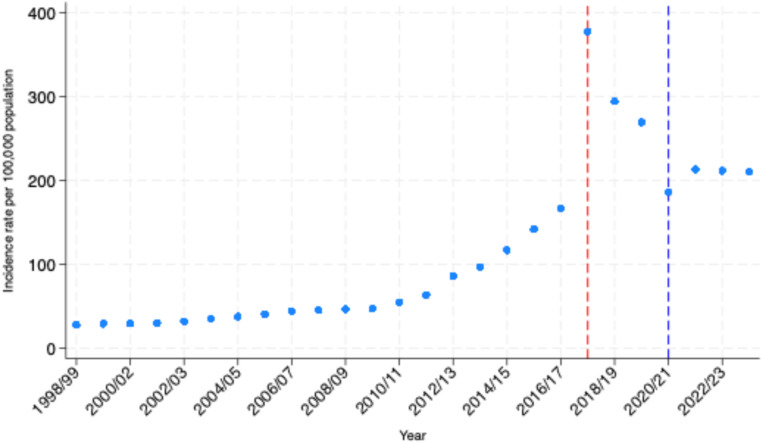
Trends in sepsis-coded hospital admissions in England (all ICD-10 codes) by year. Admissions are reported per 100,000 population of England using ONS mid-year population estimates. The dashed red line corresponds to the introduction of sepsis coding changes in England in 2017. The dashed blue line corresponds to the COVID-19 pandemic in England

**Fig. 1b:**
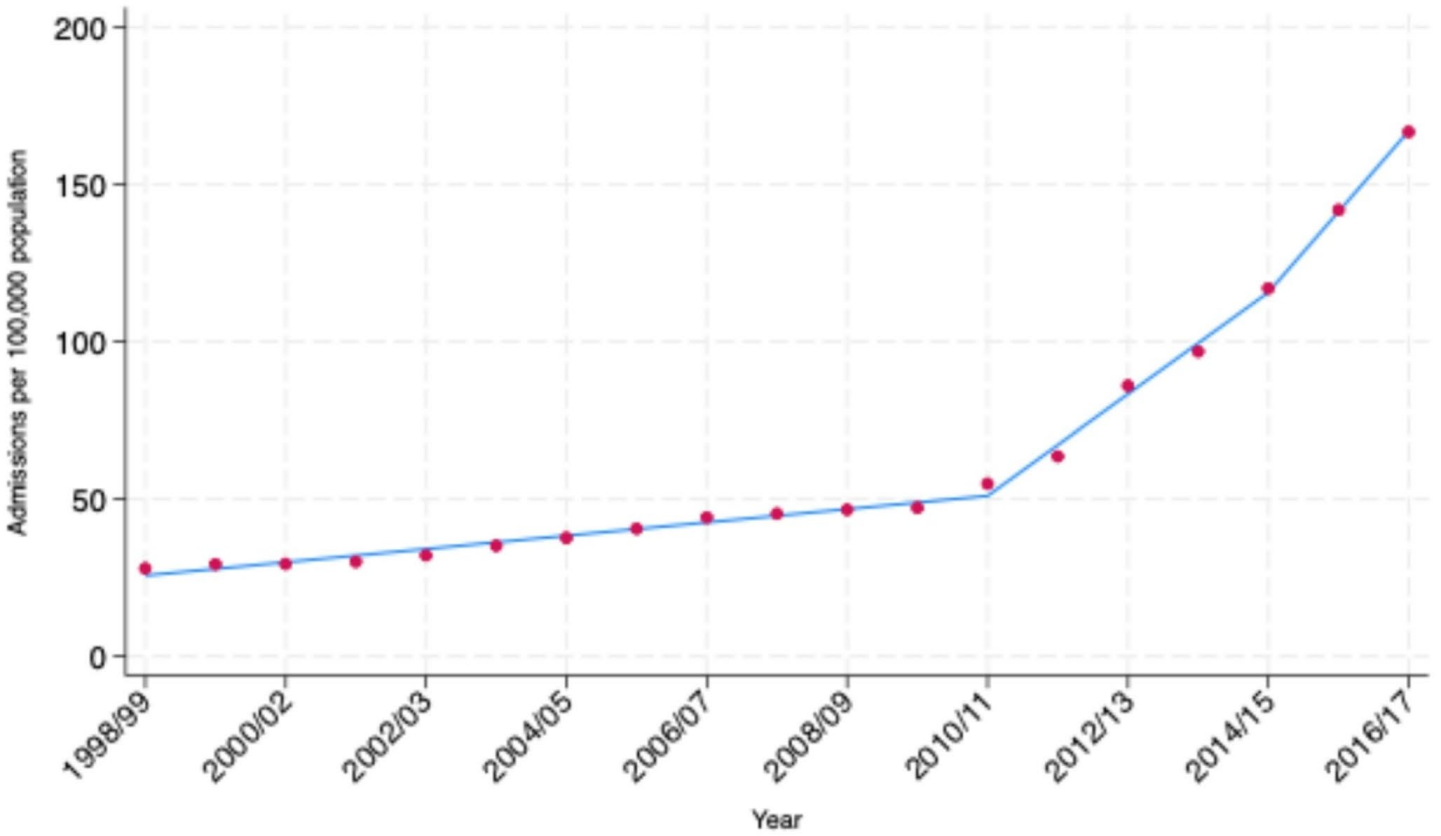
Joinpoint analysis was used to identify points where trends changed significantly. Breakpoints were detected in 2010/11 and 2014/15 indicating shifts in the rate of incidence change

Regression analysis was restricted to the years up to and including 2016 due to the impact of the coding changes described above. The incidence of hospital admissions with a primary sepsis diagnostic-code increased by 6.3 admissions per 100,000 population per year between 1998 and 2016 (95% Confidence Interval (CI): 4.5 to 8.2) Model diagnostics revealed non-linear trends in annual admission-incidence. Restricted cubic splines confirmed the gradual increase from 1998 to 2010 described above, followed by a steeper rise from 2010 to 2016. Joinpoint analysis confirmed this pattern, identifying temporal changes in the admission-incidence rate in 2010 and 2014 *(see* Fig. 1b*)*. The estimated annual increase in sepsis-coded hospital admissions was 2.1 cases per 100,000 population (95% CI: 1.8 to 2.4) prior to 2010, 16.2 cases per 100,000 population (95% CI: 15.1 to 17.2) between 2010 and 2014 and 9.5 cases per 100,000 population (95% CI: 6.0 to 13.0) post 2014.

### Incidence of sepsis-coded admissions due to specific pathogens of interest

**Fig. 2:**
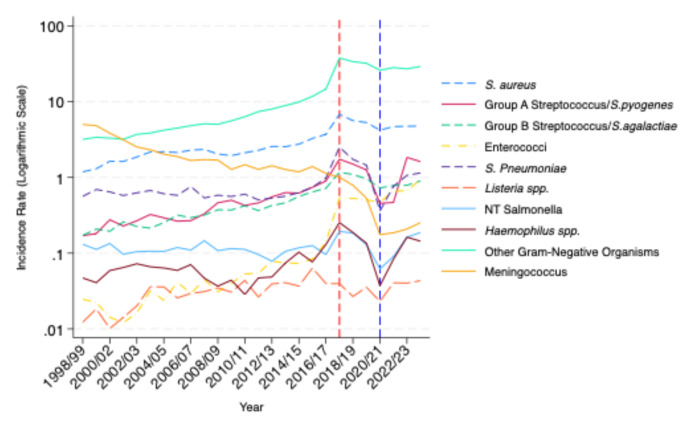
Trends in sepsis-coded hospital admissions caused by particular pathogens of interest by year. Admissions are reported per 100,000 population of England using ONS mid-year population estimates and are shown on a logarithmic scale for clarity. The dashed red line corresponds to the introduction of sepsis coding changes in England in 2017. The dashed blue line corresponds to the COVID-19 pandemic in England

Sepsis-coded admissions increased for all pathogens of interest between 1998 and 2023, except for meningococcal sepsis which saw a 20-fold decline from 5.0/100,000 admissions to 0.25/100,000 (*see* Fig. 2).

The largest increase was seen for sepsis-coded admissions due to group D streptococci and enterococci. Admissions with this code increased 47-fold from 0.02/100,000 admissions to 0.94/100,000 across the study period. Admissions due to sepsis caused by group A streptococcus increased 9.5-fold from 0.17/100,000 to 1.6/100,000. Admissions due to sepsis caused by other Gram-negative organisms increased 9-fold from 3.2/100,000 to 29.2/100,000. Admissions due to sepsis caused by *S. aureus* increased four-fold from 1.2/100,00 to 4.8/100,000. Admissions due to sepsis caused by group B streptococcus increased five-fold from 0.17/100,000 to 0.90/100,000. Hospital admissions due to pneumococcal sepsis increased two-fold from 0.56/100,000 to 1.2/100,000 and admissions due to sepsis caused by *Haemophilus spp*. increased by three-fold from 0.05/100,000 to 0.14/100,000. Hospital admissions due to sepsis caused by foodborne pathogens such as *Listeria spp* increased fourfold from 0.01/100,000 to 0.04/100,000. Admissions due to sepsis caused by non-typhoidal salmonella increased 1.5-fold from 0.13/100,000 to 0.19/100,000.

A spike in sepsis-coded hospital admissions was seen in 2017 for all pathogens except *Listeria spp*. and meningococcal sepsis. This corresponded with changes to sepsis coding in England. Admission rates then declined in 2018 following further coding changes for all pathogens except group D streptococci and enterococci.

### Impact of the COVID-19 pandemic

The HES reporting-period from April 2020 to March 2021, corresponding with the first and second waves of the COVID-19 pandemic, saw a reduction in sepsis-coded hospital admissions caused by all pathogens of interest. Sepsis-coded admissions due to enterococci and group D streptococci saw only a minor decrease of 6%. Admissions due to sepsis caused by other Gram-negative organisms, *S. aureus* and group B streptococcus decreased by 19.0% 20.7% and 24.0% respectively. Admissions due to sepsis attributed to foodborne pathogens including *Listeria spp.* and non-typhoidal salmonella saw a two-fold decrease over this period. Admissions due to sepsis caused by group A streptococcus, meningococcus, *Haemophilus spp*, and pneumococcus saw the largest falls in admission incidence. Admissions for sepsis caused by these pathogens decreased 2.8-fold, 3-fold, 3.5-fold and 4.0-fold respectively. An important caveat is that it is not possible to distinguish the impact of major changes to NHS coding guidance in 2017 and 2018 from that of the COVID-19 pandemic on these trends.

### Sepsis mortality rates

**Fig. 3:**
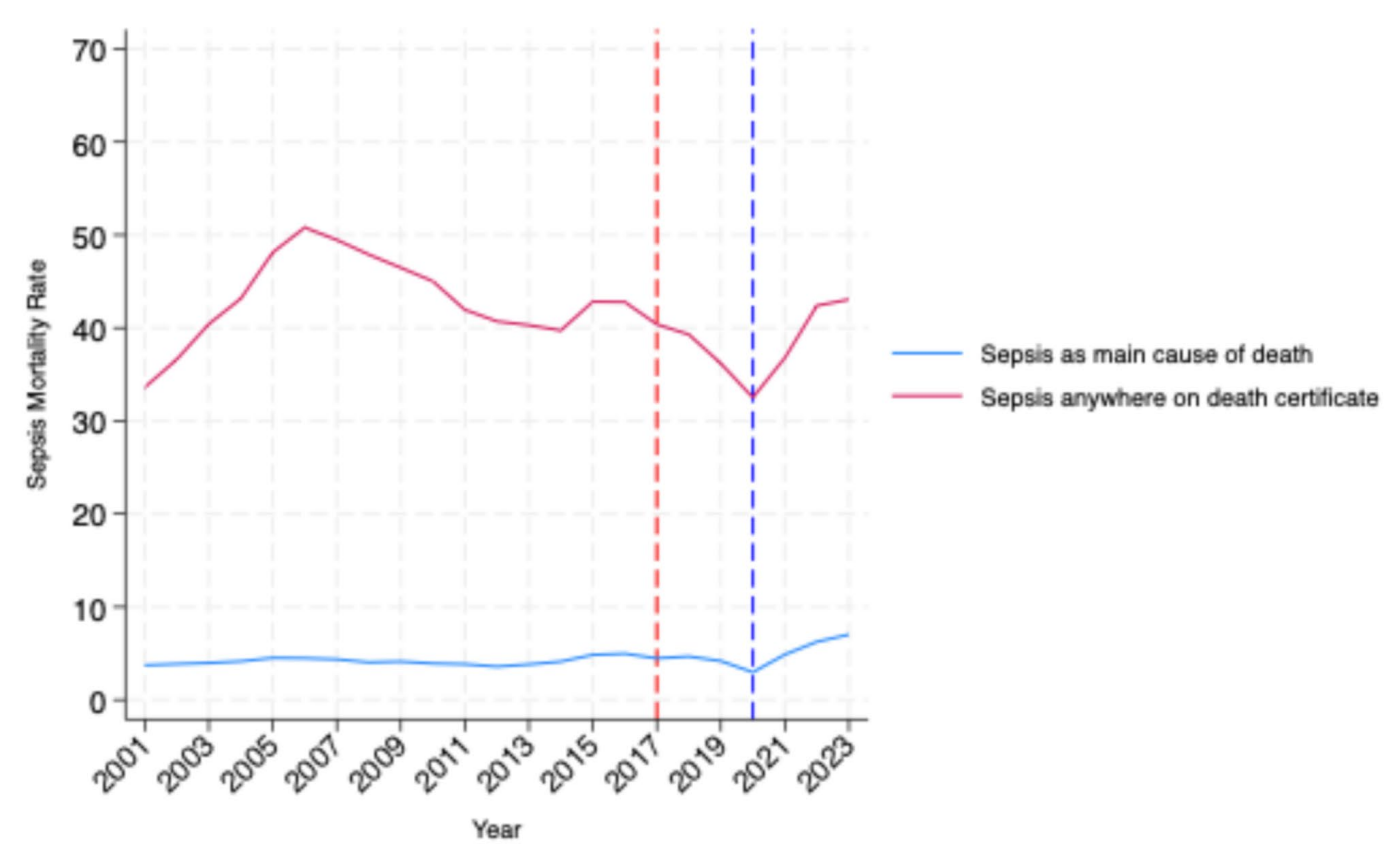
Death registrations in England and Wales with sepsis as the main cause of death or mentioned anywhere on the death certificate. Deaths are reported per 100,000 population of England and Wales using ONS mid-year population estimates. The dashed red line corresponds to the introduction of sepsis coding changes in England in 2017. The dashed blue line corresponds to the COVID-19 pandemic in England

Death registrations where sepsis was mentioned anywhere on the death certificate increased by 51.2% from 33.6 per 100,000 in 2001 to 50.8 per 100,000 in 2006 *(see* Fig. 3*)*. There was then a gradual decline to 39.7 per 100,000 by 2014. The death rate then remained relatively stable with small fluctuations before dropping sharply to 32.5 per 100,000 in 2020, coinciding with the first and second waves of the COVID-19 pandemic. Death rates then rebounded to pre-pandemic levels of 42.4 per 100,000 by 2022. Death registrations where sepsis was the main, underlying diagnosis on the death certificate have been stable with a gradual increase since 2020.

### Age and sex distribution

**Fig. 4:**
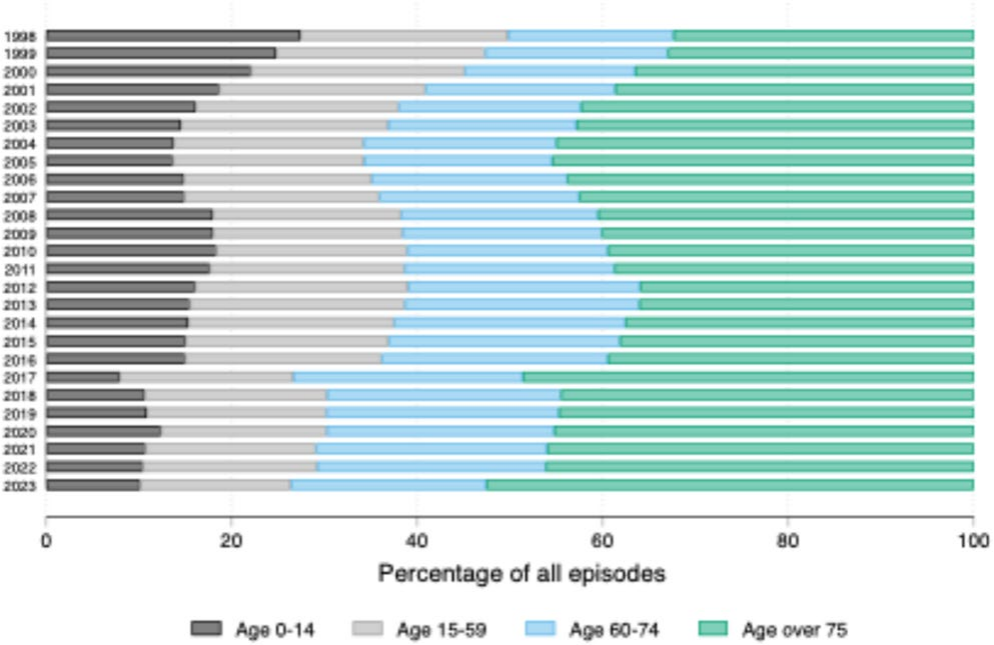
Proportion of patients coded as admitted with sepsis aged 0–14 years, 15–59 years, 60–74 years and ≥ 75 years

Sepsis increased across all age groups over the study period. The most marked increase was observed in sepsis event rates in patients aged ≥ 75 years from 32.4 to 52.6%. Sepsis in children aged ≤ 14 years increased, although declined as a proportion of the overall cases from 27.4 to 10.2% (*see* Fig. 4). The mean age increased across the study period from 34.9 years in 1998 to 45.9 years in 2023. There was a higher proportion of male patients than female patients across all years with a mean of 56.2%.

### Median length of stay

Mean median length of stay for hospital admissions due to sepsis was stable across the study time-period, being 6.8 days in 1998 and 6.6 days in 2023. The mean median length of stay was 6.1 days across all years. In 2023 sepsis contributed > 800,000 hospital bed days, compared to < 100,000 in 1998.

## Discussion

In this study we demonstrated that sepsis-coded hospital admissions increased 7.5-fold from 27.9 admissions per 100,000 population in 1998 to 210.4 admissions per 100,000 population in 2023. Sepsis admissions increased as a proportion of all-cause hospital admissions over the same time-period. This observation corroborates previously published data from English ICUs that demonstrated a rising incidence of sepsis between 1996 and 2004 [13] and 2011 to 2015 [5]. Our results align with the findings of studies from Europe, Australia and New Zealand and the United States which have demonstrated rising rates of sepsis [6–9, 22]. We found that sepsis-coded admissions did not increase at a constant rate but displayed a complex pattern with periods of varying acceleration and decline. The most pronounced increases occurred between 2010 and 2014 and in 2017.

Changes to coding methodology may have had a major impact on the incidence of sepsis-coded admissions described in this study. Specifically, an apparent spike in hospital admissions due to sepsis was observed in 2017 for all pathogens except *Listeria spp*. and meningococcal sepsis. This coincided with NHS Digital-mandated changes to sepsis diagnostic coding guidance in April 2017 that aimed to increase the identification of sepsis as the primary admission diagnosis [23]. Further guidance was issued in 2018 allowing local hospital coding departments a greater level of discretion and acknowledging that the previous guidance had led to a probable over-reporting of sepsis [24]. Following these adjustments, our study found that hospital admissions declined in 2018 for most pathogens, except group D streptococci and enterococci. Previous research using HES data also found that hospital admissions in England with a primary diagnosis code for sepsis increased significantly after the 2017 coding changes, followed by a decline in 2018, although admission rates remained significantly higher than in pre-2017 [25].

Changes to the definition of sepsis over the study time-period may also have affected recorded incidence of sepsis-coded admissions. There have been three major updates to the definition of sepsis, in 1991 [26], 2001 [27] and 2016 [1]. The gradual increase in sepsis-coded hospital admissions observed from the early 2000s, as well as the sharper rise seen in 2017, may have been impacted by the introduction of the new definitions in 2001 and 2016.

The introduction of sepsis prevention programmes, for example the Surviving Sepsis Campaign, may have impacted the recording of hospital admissions due to sepsis. The Surviving Sepsis Campaign guidelines were first published in 2004 [28], were updated in 2008 [29] and 2012 [30], and again most recently in 2021 [12]. Such initiatives may have facilitated improved recognition of sepsis within emergency departments leading to the increases in diagnostic coding observed in this study. The guideline updates in 2008 and 2012 may also have contributed to the pronounced rise in sepsis-coded hospital admissions between 2010 and 2014 demonstrated in this study.

One factor that may be contributing to the rising incidence of sepsis-coded hospital admissions in England, aside from changes to coding practices and definitions, is an ageing population. This study demonstrated a change in the age-distribution of sepsis-coded admissions over time, with a rising incidence amongst patients aged over 75 years. Of note, the number of people aged over 65 years in England and Wales increased by approximately 20% between 2011 and 2021 demonstrating a demographic shift over the study time period [31]. A further contributing factor may be rising usage of immunosuppressive therapies for autoimmune diseases and cancers, conditions which have increased in prevalence over the study period [32, 33].

Another possible contributor to rising rates of sepsis-coded admissions in England is antimicrobial resistance (AMR). Recent data from UKHSA show that antimicrobial resistant infections are increasing in England, with the majority caused by *E. coli* [34]. Rising rates of AMR are likely to lead to an increase in sepsis as infections become resistant to first-line antibiotics and more difficult to treat. One study from the United States found that state-specific prevalence of resistance to several antibiotics in different bacteria is positively correlated with rates of hospitalization with septicaemia and sepsis-mortality in multiple age groups of adults [34]. A study from Taiwan found that multidrug resistance was associated with the development of severe sepsis or septic shock among patients with bacteraemic urinary tract infections [35].

The second main finding from our study was that sepsis-coded admissions caused by most pre-specified pathogens of interest rose across the study time-period. The UKHSA collects surveillance data on bacteraemias in England due to various pathogens of interest [36]. Bacteraemia is a diagnosis that is closely related to sepsis. Although not all patients admitted with sepsis will have a proven bacteraemia, it is helpful to compare trends in surveillance data with data on hospital admissions due to sepsis. An important caveat to our results is that new microbiological diagnostic techniques have been introduced over the time-period of this study, for example matrix-assisted laser desorption ionization time-of-flight mass spectrometry (MALDI-TOF) [37, 38]. The implementation of MALDI-TOF and other new technologies may have impacted pathogen identification which could have influenced the results of this study.

Our study found a significant increase in sepsis-coded admissions caused by enterococcal *spp* over time. Bacteraemia surveillance data from UKHSA demonstrate a 63.5% rise in bacteraemia due to enterococcal *spp* across the decade from 2013 to 2023 with the highest rates reported in those aged over 75 years [39]. UKHSA surveillance data also corroborate the rise in sepsis-coded admissions due to *S. aureus* shown in this study with the incidence of *S. aureus* bacteraemia (SAB) increasing 41.6% between 2011 and 2024 [36].

Surveillance data demonstrate a rise in bacteraemias due to Gram-negative bacteria including *E. coli* and *Klebsiella spp* across the study time-period in keeping with our findings of rising hospital admissions due to Gram-negative sepsis [36]. Sepsis due to “other Gram-negative organisms” was the commonest pre-specified ICD-10 code of interest associated with a hospital admission in this study. Whilst this was in keeping with studies of patients with sepsis admitted to intensive care units internationally [40–42], it is important to note that this code includes multiple different species of bacteria compared to the other codes which are single species only.

The systematic implementation of vaccination programmes over the study period may have affected the incidence of sepsis-coded admissions. Admissions due to meningococcal sepsis were unique among the pre-specified pathogens of interest as they declined over the study period. This was likely driven by the introduction of vaccines against meningococcus in England. A vaccine against group C was introduced in 1999, an infant MenB vaccine was introduced in 2015, along with a teenage programme against meningococcal A, C, W and Y, also in 2015 [43]. This finding reinforces the positive impact of vaccination campaigns despite increasing vaccine-hesitancy.

The third main finding from this study is that sepsis-coded hospital admissions have returned to the pre-pandemic trend despite a decrease in the period from April 2020 to March 2021, coinciding with the first and second waves of the COVID-19 pandemic. The largest decreases were seen for sepsis due to pathogens spread via droplets or close contact including group A streptococcus, meningococcus, *Haemophilus spp*, and pneumococcus. This is likely due to the impact of social distancing measures. Sepsis-coded admissions due to foodborne pathogens also decreased. This is in keeping with previous research published by the UKHSA that demonstrated a decrease in gastrointestinal illness activity during the COVID-19 pandemic [44] This may have been due to changes in behaviour, for example social distancing measures leading more people to prepare food at home. While discussing these trends, it is important to note that recorded hospital admissions due to sepsis had been declining since 2018, possibly driven by the coding change described above. An important caveat is that any data collected during the COVID-19 pandemic must be interpreted with caution due to the extraordinary pressures on the NHS and other governmental institutions at that time. The return to the pre-pandemic trend in sepsis admissions highlights the continued importance of sepsis prevention measures.

The fourth key finding in our study was that sepsis-related mortality has returned to pre-pandemic levels after decreasing sharply during 2020. The decrease in sepsis-related mortality may have been artefactual related to the competing risks of COVID-19, due to a reduction in the incidence of sepsis or increasing deaths due to COVID-19. Our study also corroborates the decline in sepsis-related mortality in England in recent decades observed in other studies using national data from hospital admissions [13] and ICU admissions [45, 46]. This decline in sepsis-related mortality occurred on a background of rising sepsis-coded hospital admissions. It is not possible to conclusively explain the underlying reasons behind the discrepancy between recorded admission-incidence and mortality. One possible explanation may be that the rise in coded hospital admissions reflects coding changes and improved recognition of sepsis rather than a rise in clinically severe sepsis. Another possible factor may be that the introduction of initiatives including the Surviving Sepsis Campaign [12], may have led to improvements in the clinical management of sepsis in hospitals that have reduced mortality.

The final important finding in our study was that sepsis contributed > 800,000 hospital bed days in 2023, compared to < 100,000 in 1998. Many cases of sepsis will require an admission to an ICU which is significantly more expensive than a standard ward. Recent data from the British government suggest that an intensive care bed costs the NHS £1881 per night compared to £901 for a standard non-elective bed [47]. Rising bed-days due to sepsis admissions and the high costs involved are likely to place a significant financial burden on the NHS. The mean median length of stay was 6.1 days across the study time-period. This is shorter than in other previously published British studies from intensive care units [45]. This likely reflects the broader range of disease severity represented in our study which captured data from all hospital admissions, including those to general hospital wards. British data suggest that only 16% of sepsis cases are referred to critical care [48]. Patients with sepsis admitted to critical care in England are likely to be those with the most severe disease who will have more complex and longer recovery trajectories than many patients admitted elsewhere.

This study has several strengths. We used population-level, aggregate datasets from NHS Hospitals, accounting for 98–99% of hospital activity in England [16]. This gives a very large sample size allowing us to draw inferences about rarer causes of sepsis such as *Listeria spp*. and non-typhoidal Salmonella. It also provides insights into trends across the whole of England over a 25-year period. The HES data analysed in this study is derived from diagnostic coding performed by hospital trusts. This process is audited and has been demonstrated to be sufficiently robust for use in research [17].

Our study also has limitations. Two major updates to the definition of sepsis occurred during the study period [1, 27]. These changes are likely to have impacted clinical decision-making around whether individual cases met the diagnostic criteria for sepsis. Likewise, rising awareness and recognition of sepsis amongst healthcare professionals due to awareness campaigns [12] may also have led to an increase in diagnoses. These practice changes may have affected coding outcomes as trust coding departments rely on medical notes for correct attribution of ICD-10 codes. Changes to NHS-mandated coding guidance in 2017 and 2018 will also have impacted recorded rates of sepsis-coded admissions. These factors reflect the limitations, and possible imprecision, of relying on administrative data for epidemiological analysis. Without patient-level data it is not possible to objectively confirm whether the label of “sepsis” has been correctly attributed in all cases. Despite these limitations, the concurrent rise in many bacteraemias described by UKHSA over the study period [36], supports the conclusion that there may have been a genuine rise in sepsis cases over the study period. Data from other healthcare settings indicate that ICD-10 codes in administrative data have relatively low sensitivity but high specificity for identifying pathogen-specific infections and sepsis [49, 50]. This suggests that while ICD-10 codes may identify pathogen-specific infections correctly, cases may be missed, limiting the validity of using coding to capture the full burden of pathogen-specific sepsis cases. This suggests that our study may have underestimated the burden of sepsis in England. There are no validation studies assessing the validity of pathogen-specific sepsis codes in HES-APC, highlighting an important evidence gap for future research.

A further limitation is that it was not possible to explore sepsis severity. Changes to the use and relative positions of codes for septic shock (R57.2) and organ dysfunction (R65.2) within the NHS over time meant that it was not possible to perform a meaningful analysis of temporal trends for these codes. Due to the aggregate nature of the data, it was not possible to explore the impact of individual, patient-level factors including co-morbidities on hospital admissions due to sepsis. A further limitation is that we do not have individual-level data on outcomes of patients admitted with sepsis although we have been able to analyse population-level data on sepsis mortality in England and Wales provided by the ONS across a similar time-period. Both hospital admissions due to sepsis in England and overall sepsis mortality in England and Wales declined in 2020 but due to the aggregate nature of the HES and ONS death data it is not possible to comment on the case-fatality rate of those admitted with sepsis in this period. This was noted to be higher in a large Danish study [14]. We also did not have data on how the diagnosis of sepsis due to a particular pathogen of interest was defined. Microbiological confirmation for admissions coded as due to pathogen-specific sepsis were not available in this administrative dataset. We were therefore unable to confirm whether these diagnostic codes were correctly attributed. This may have decreased the reliability of our findings.

Our study shows that the recorded incidence of sepsis-coded hospital admissions has increased in England over recent years. This is true for most of the bacterial pathogens studied. Hospital admissions due to sepsis caused by meningococcus declined, likely due to the success of vaccination campaigns. Although coding changes and improved recognition of sepsis may have contributed to rising sepsis diagnoses, the true burden of sepsis in England may be rising. Sepsis-coded admissions and sepsis-related mortality decreased during the COVID-19 pandemic but have since returned to the pre-pandemic trend. Further research is needed to monitor the trends over the coming years. An aging population, more invasive procedures and increasing levels of immunosuppression are likely to lead to a greater at-risk population. Further research into sepsis epidemiology using individual patient-level data and research into predicting and preventing sepsis is urgently needed.

## Electronic supplementary material

Below is the link to the electronic supplementary material.

Supplementary Material 1

## Data Availability

Hospital episode data were obtained from the Hospital Admitted Patient Care Activity Records. These are available from: https://digital.nhs.uk/data-and-information/publications/statistical/hospital-admitted-patient-care-activity.Mid-year population estimates for England were used in the analysis of hospital admissions. These are published by the Office for National Statistics (ONS) and are available at: https://www.ons.gov.uk/peoplepopulationandcommunity/populationandmigration/populationestimates/datasets/estimatesofthepopulationforenglandandwales.Data on death registrations due to sepsis used data from England and Wales published by the ONS. These are available at https://www.ons.gov.uk/peoplepopulationandcommunity/birthsdeathsandmarriages/deaths/adhocs/2141deathsinvolvingsepsisenglandandwales2001to2023.
