# Supplementary material for: Rising rates of sepsis in England: an ecological study
Source: Infection. 2025 Jul 15;53(6):2601–12. doi: 10.1007/s15010-025-02601-0 (PMC12675601; doi:10.1007/s15010-025-02601-0)
Supplement: Supplementary file 1 — Supplementary Material 1 [file 15010_2025_2601_MOESM1_ESM.docx]

# **Rising Rates of Bacterial Sepsis in England: An Ecological Study**

Victoria B Allen ^a^

Mark D Russell ^a^

Katie Bechman ^a^

Maryam A Adas ^a^

Anna L Goodman ^b, c, d^

Mark J McPhail ^e^

Sam Norton ^a^

James B Galloway ^a^

^a^ Centre for Rheumatic Diseases, King’s College London, London, UK

^b^ Department of Infection, King’s College London, UK

^c^ Department of Infection, Guy’s and St Thomas’ NHS Foundation Trust, London UK

^d^ MRC Trials Unit, University College London, London, UK

^e^ Institute of Liver Studies, King’s College London, London UK

Victoria.1.allen@kcl.ac.uk

**Fig 1. Relative change in hospital admissions due to sepsis for all included ICD-10 codes compared to previous year. The dashed red line corresponds to the introduction of sepsis coding changes in England in 2017.**

The dashed blue line corresponds to the COVID-19 pandemic in England.

**Pre-Specified ICD-10 Codes:**

A02.1 Salmonella sepsis

A20.7 Septicaemic plague

A22.7 Anthrax sepsis

A24.1 Acute and fulminating melioidosis

A26.7 Erysipelothrix sepsis

A32.7 Listerial sepsis

A39.4 Meningococcaemia, unspecified

A40.0 Sepsis due to streptococcus, group A

A40.1 Sepsis due to streptococcus, group B

A40.2 Sepsis due to streptococcus, group D and enterococcus

A40.3 Sepsis due to Streptococcus pneumoniae

A40.8 Other streptococcal sepsis

A40.9 Streptococcal sepsis, unspecified

A41.0 Sepsis due to Staphylococcus aureus

A41.1 Sepsis due to other specified staphylococcus

A41.2 Sepsis due to unspecified staphylococcus

A41.3 Sepsis due to Haemophilus influenzae

A41.4 Sepsis due to anaerobes

A41.5 Sepsis due to other Gram-negative organisms

A41.8 Other specified sepsis

A41.9 Sepsis, unspecified

A42.7 Actinomycotic sepsis

B00.7 Disseminated herpesviral disease

B37.7 Candidal sepsis

O75.3 Other infection during labour, Sepsis during labour

O85 Puerperal sepsis

P36 Bacterial sepsis of newborn

P36.0 Sepsis of newborn due to streptococcus, group B

P36.1 Sepsis of newborn due to other and unspecified streptococci

P36.2 Sepsis of newborn due to Staphylococcus aureus

P36.3 Sepsis of newborn due to other and unspecified staphylococci

P36.4 Sepsis of newborn due to Escherichia coli

P36.5 Sepsis of newborn due to anaerobes

P36.8 Other bacterial sepsis of newborn

P36.9 Bacterial sepsis of newborn, unspecified

R65.1 Systemic Inflammatory Response Syndrome of infectious origin with organ failure

A39.1 Waterhouse-Friderichsen syndrome

A39.2 Acute meningococcaemia

A39.3 Chronic meningococcaemia

A48.3 Toxic shock syndrome

R57.2 Septic shock
